# Supplementary material for: Development and Immunogenicity Study of Subunit Vaccines Based on Spike Proteins of Porcine Epidemic Diarrhea Virus and Porcine Transmissible Gastroenteritis Virus
Source: Vet Sci. 2025 Feb 1;12(2):106. doi: 10.3390/vetsci12020106 (PMC11860644; doi:10.3390/vetsci12020106)
Supplement: Supplementary file 1 [file vetsci-12-00106-s001.zip › vetsci-3386539-supplementary.pdf]

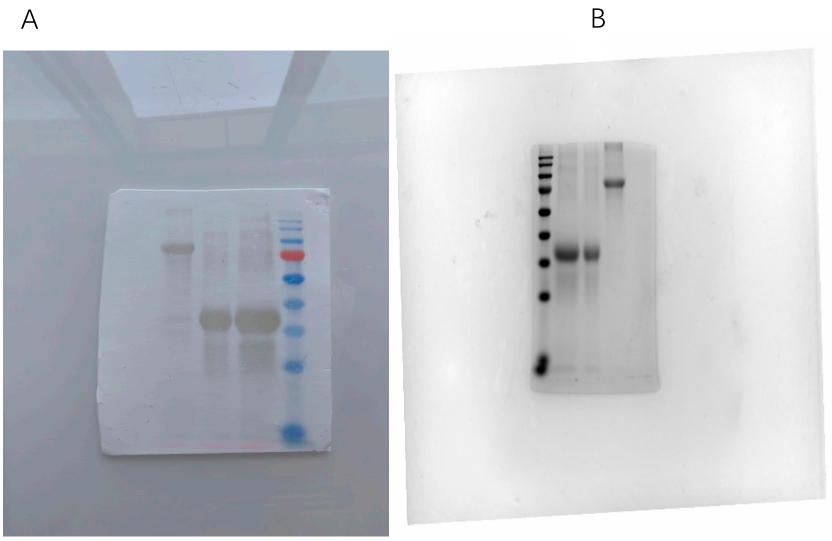


**Figure S1.** Expression and purification of recombinant proteins. **(A)** The expression of the PEDV S1, TGEV S1 and PEDV S1-TGEV S1 recombinant proteins was confirmed by SDS-PAGE with coomassie brilliant blue staining. Lane M: Protein marker; Lane 1: PEDV S1 recombinant protein; Lane 2: TGEV S1 recombinant protein; Lane 3: PEDV S1-TGEV S1 recombinant protein. **(B)** The expression of the PEDV S1, TGEV S1, and PEDV S1-TGEV S1 recombinant proteins was confirmed by Western blotting analysis using anti-PEDV S and anti-TGEV polyclonal antibodies. Lane M: Protein marker; Lane 1: PEDV S1 recombinant protein; Lane 2: TGEV S1 recombinant protein; Lane 3: PEDV S1-TGEV S1 recombinant protein.
